# Identification of coral spawn source areas around Sekisei Lagoon for recovery and poleward habitat migration by using a particle-tracking model

**DOI:** 10.1038/s41598-021-86167-5

**Published:** 2021-03-26

**Authors:** Naoya Takeda, Motohiko Kashima, Sachika Odani, Yusuke Uchiyama, Yuki Kamidaira, Satoshi Mitarai

**Affiliations:** 1 Faculty of Humanities and Sciences, Kobe Gakuin University, Kobe, Japan; 2 Department of Civil Engineering, Kobe University, Kobe, Japan; 3 Nuclear Science and Engineering Center, Japan Atomic Energy Agency, Tokai, Japan; 4 Marine Biology Unit, Okinawa Institute of Science and Technology, Onna, Japan

**Keywords:** Ocean sciences, Marine biology, Physical oceanography, Environmental sciences, Climate change, Ocean sciences, Marine biology, Physical oceanography

## Abstract

A massive coral bleaching event occurred in 2016 in the interior of Japan’s largest coral lagoon, the Sekisei Lagoon, located in the Kuroshio upstream region in southwestern Japan. Recovery of the coral lagoon will require the influx of coral spawn and larvae; therefore, it is important to identify and conserve source sites. A surface-particle-tracking simulation of coral spawn and larvae was used to identify source areas of coral spawn outside of the Sekisei Lagoon for potential recovery of the interior lagoon. The northern coastal zone of Iriomote Island, including Hatoma Island, was identified as a major source area. Hatoma Island was also identified as a key source for the Kuroshio downstream region and for aiding the poleward migration of coral habitat under ongoing global climate change, making it one of the most important source areas in the Nansei Archipelago.

## Introduction

**Figure 1:**
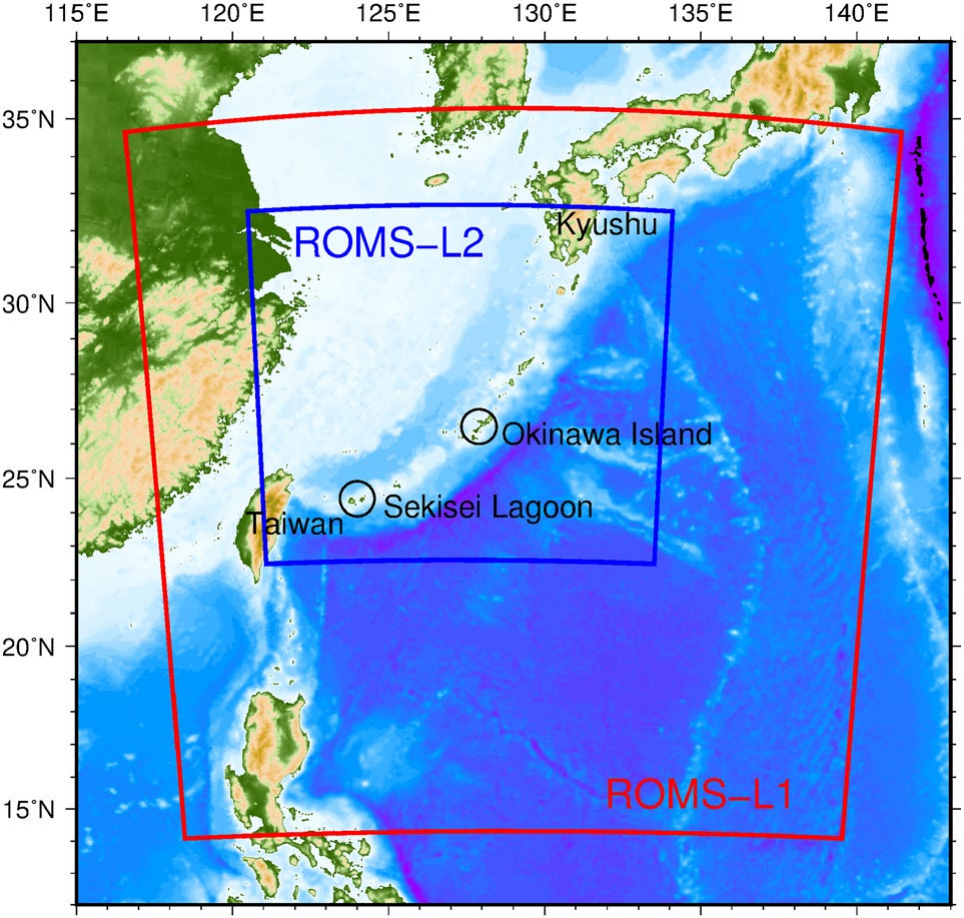
Model domains for ROMS-L1 (red) and ROMS-L2 (blue). Colors indicate bathymetry.

Corals are an integral part of the coral reef ecosystem and are also a major tourism resource in the Nansei Archipelago, a string of islands in southwestern Japan located between Kyushu and Taiwan (Fig. 1). Coral reefs around Okinawa Island, which is in the middle of the archipelago, were massively damaged by a coral bleaching event in 1998^1,2^. Direct or inter-generational gene flows link coral populations across the Nansei Archipelago^3,4^.

The Sekisei Lagoon is the largest coral lagoon in Japan and is located in the Kuroshio upstream region (i.e., the southern Nansei Archipelago). The lagoon is thought to help maintain coral reef ecosystems across the entire archipelago by supplying coral spawn and larvae to downstream sites (i.e., relatively northern regions of the archipelago). However, corals in this lagoon were impacted considerably by a massive bleaching event in 2016, some 18 years after the 1998 event that hit Okinawa Island. Both bleaching events (1998 and 2016) occurred during the years of GCBE (Global-scale Coral Bleaching Events)^5^. Diving surveys showed that of the 11 major coral species in the lagoon, the rate of colony bleaching or death exceeded 98% in 10 species^6^. Coral reefs regenerate through the influx of coral spawn and larvae especially from upstream regions; this means that the recovery of corals in the Sekisei Lagoon is a necessary first step toward the recovery of the entire Nansei Archipelago and for aiding poleward migrations of coral habitat under the influence of ongoing global climate change.

Quantitative pathways of coral spawn and larvae dispersal through direct advection by ocean currents and associated dispersal effects were investigated in our previous study, which used the Regional Oceanic Modeling System (ROMS) coupled with a 3-D Lagrangian particle-tracking model to simulate coral spawn and larvae as neutrally buoyant particles. Although the study showed that particles released off the Yaeyama Islands (i.e., the region around the Sekisei Lagoon) may be advected more than 400 km northeast to reach Okinawa Island within a typical pelagic larval duration of 21 days^4^, we were not able to identify specific source areas for the coral spawn and larvae. Therefore, our current study aims to identify major source areas for (1) the interior Sekisei Lagoon, and (2) the entire Kuroshio downstream region, (and hence, for aiding the poleward coral habitat migration) by using 3-D Lagrangian particle-tracking simulations^4^.

## Methods

We used submesoscale eddy-resolving synoptic ocean models based on the Regional Oceanic Modeling System (ROMS) in a double-nested configuration coupled with a 3-D Lagrangian particle-tracking model embedded in the Japan Coastal Ocean Predictability Experiment 2 (JCOPE2)^4^. JCOPE2 is a numerical reanalysis product for the northwestern Pacific Ocean assimilated with a vast amount of satellite and in situ data^7^. ROMS-L1 (Fig. 1, red rectangle) has a spatial resolution of 3 km and a calculation period of 1 January 2005 to 2 November 2015. ROMS-L2 (Fig. 1, blue rectangle) has a higher spatial resolution of 1 km and a shorter calculation period of 27 December 2010 to 2 November 2015^4^ (Table 1).

**Table 1:** Model parameters for ROMS-L1 and ROMS-L2 setting conditions^4^.

	ROMS-L1	ROMS-L2
Computational period	1 Jan 2005–2 Nov 2015	27 Dec 2010–2 Nov 2015
Grid cells	768 × 768 (× 32 Layers)	1280 × 1280 (× 32 layers)
Horizontal grid resolution	3 km	1 km
Baroclinic time step	240 s	40 s
Surface wind stress	QuikSCAT-ECMWF (daily, until 31 Dec 2007)	JMA GPV-MSM (h)
	JMA GPV-GSM (daily, 1 Jan 2008 and later)	
Surface flux	NOAA COADS (monthly climatology)	
SST and SSS to restore	JCOPE2 (20-days averaged)	
Yangtze River discharge	Monthly climatology^11^	
Open boundary/initial conditions	JCOPE2 (daily)	ROMS-L1 (daily)
Temperature-salinity nudging	JCOPE2 (10-days averaged)	Not used
Tides	Not Used	TPXO 7.0
Topography	SIO SRTM30_Plus	

**Figure 2:**
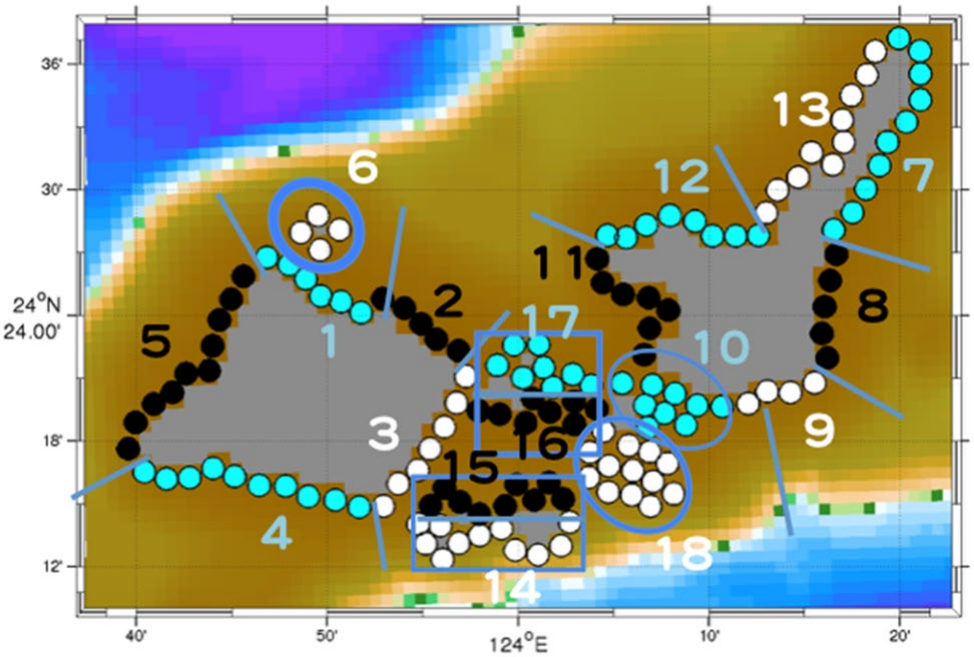
Close-up of the model domain around Sekisei Lagoon. Circles indicate source and destination areas separated into numbered groups. Groups 3, 10, and 14–18 are located in the interior and the remainder are outside of the lagoon.

To identify major source areas and common transport routes of coral spawn and larvae, about 29,000 virtual surface Lagrangian particles were tracked between 145 areas that served as both potential source areas and destination areas on land grids (Fig. 2) or on the boundaries of the ROMS-L2 model domain for at least 21 days with a time step of 200 s^8,9^. Connectivity was calculated by using probability density functions (PDFs)^10^; areas with the highest probability density were identified as major sources. Lagrangian particles were discharged at 20:00 on the first night after the full moon during a spring tide in May, which is when mass coral spawning is most likely to occur, and continued each night for the next 7 days for simulations of the Sekisei Lagoon and the next 14 days for simulations of the entire Kuroshio downstream region during the years 2012–2015.

Coastal areas around the Sekisei Lagoon (Iriomote Island, the Sekisei Lagoon, and Ishigaki Island) were divided into 18 groups (Fig. 2). Groups 3, 10, and 14–18 are located in the interior and the remainder are outside of the lagoon. All areas were considered as potential sources and as potential destinations. By using PDFs, we identified major source areas outside of the lagoon that could contribute to recovery of the interior lagoon.

## Results

### Identification of major source areas outside of the lagoon that could supply the interior lagoon

**Figure 3:**
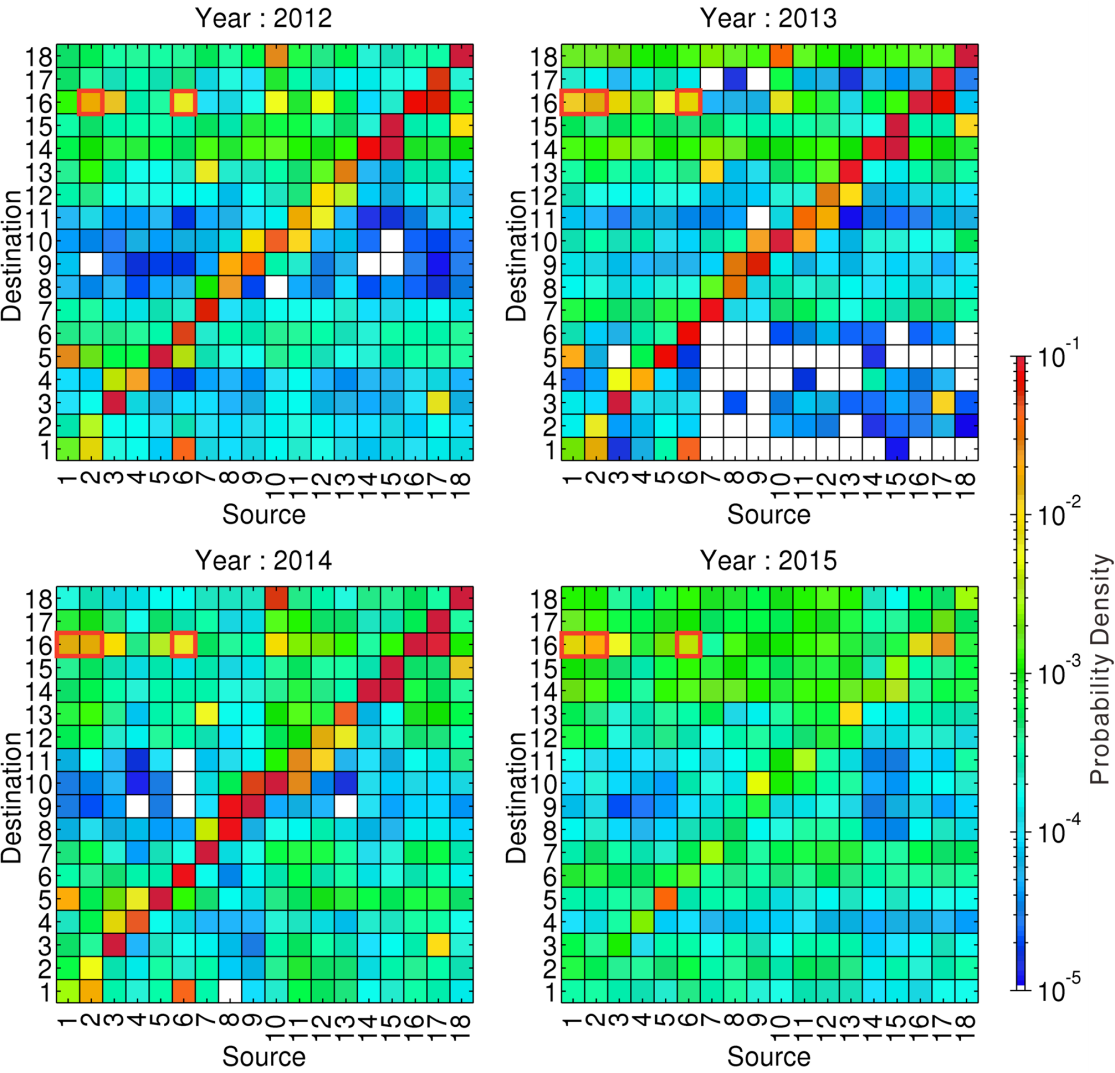
Connectivity matrices around the Sekisei Lagoon for each year during the period 2012–2015. Numbers on the abscissa (source areas) and ordinate (destination areas) correspond to the groups shown in Fig. 2. High probability densities (warm colors) indicate that numerous particles drifted from the source area to the destination area. Red outlines show high-probability-density source areas outside of the lagoon (i.e., other than groups 3, 10, and 14–18) for Group 16.

**Figure 4:**
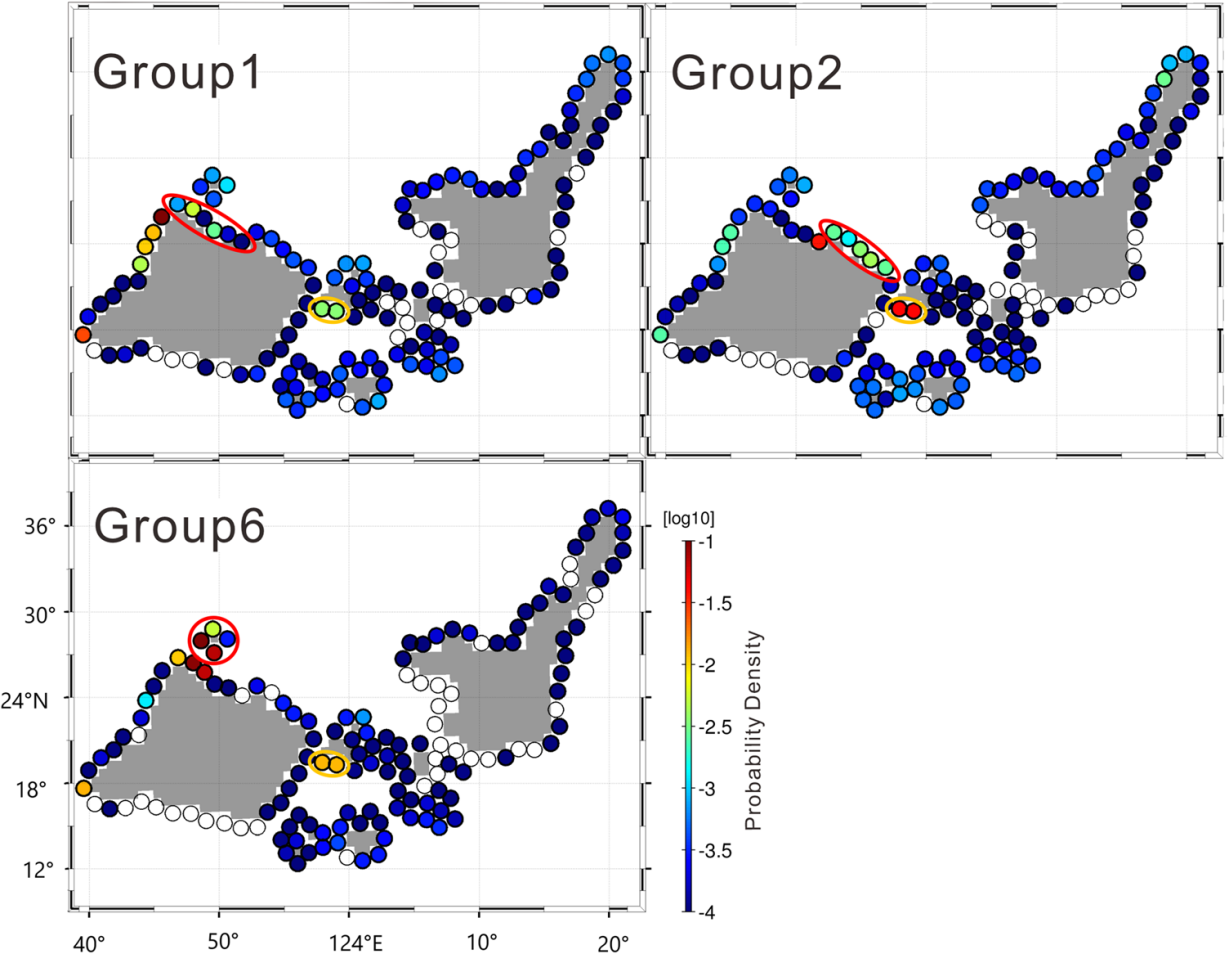
Reachability of particles from outside of the lagoon. Groups 1, 2, and 6 are indicated by red ovals, respectively. Orange ovals indicate the southern coast of Kohama Island (the western part of Group 16), which was the highest-probability destination area in the interior lagoon.

Group 16, on the southern coast of Kohama Island (Fig. 2), was the highest-probability destination area in the interior lagoon in each examined year (Figs. 3, 4). Relatively more particles drifted from the northern coastal zone of Iriomote island, especially from the northern and northeastern coasts of Iriomote Island (Groups 1 and 2, respectively) and the coast of Hatoma Island (Group 6) (Fig. 4).

**Figure 5:**
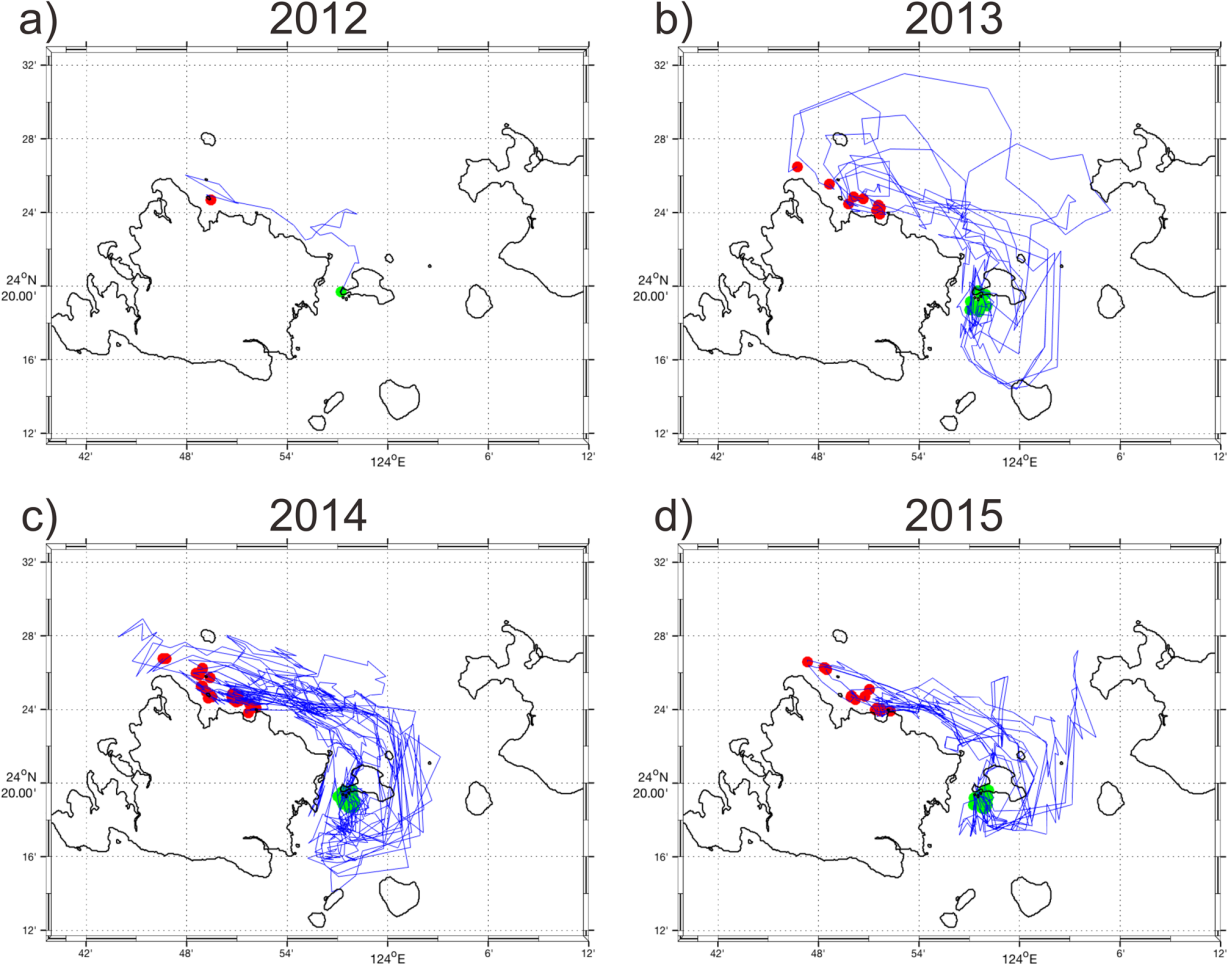
Trajectories of particles discharged from the northern coast of Iriomote Island (Group 1) that reached the southern coast of Kohama Island (Group 16) during the years 2012–2015 (a–d). Only 10% of the simulated trajectories are shown for clarity. Red and green dots indicate particle discharge and destination points in source and destination areas, respectively.

**Figure 6:**
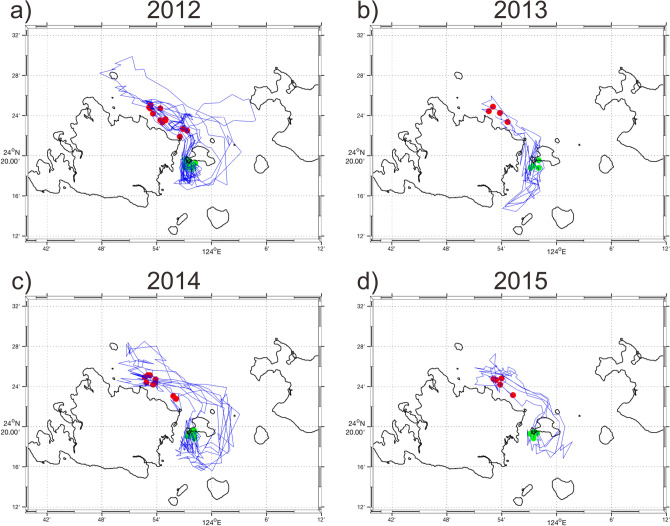
Same as Fig. 5, but from the northeastern coast of Iriomote Island (Group 2).

**Figure 7:**
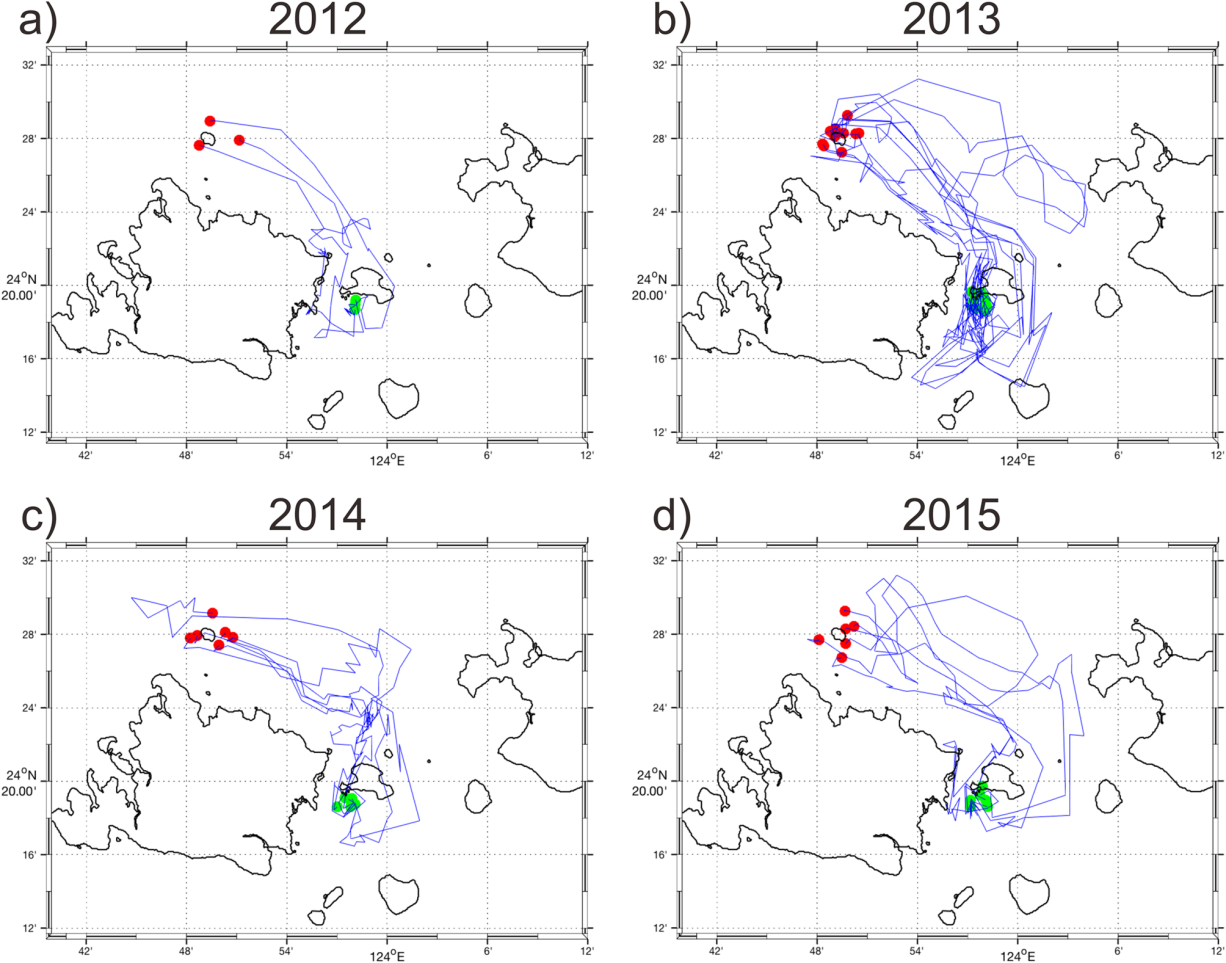
Same as Fig. 5, but from Hatoma Island (Group 6).

Two major routes toward Group 16 from Groups 1, 2, and 6 were revealed from the particle drift trajectories: one passes southward through the Yonara Channel on the west side of Kohama Island, and the second passes through the channel on the east side (Figs. 5, 6, 7). No particles drifted around Ishigaki or Iriomote islands to reach the interior lagoon.

### Identification of major source areas around the Sekisei Lagoon for the Kuroshio downstream region and for poleward coral habitat migration

**Figure 8:**
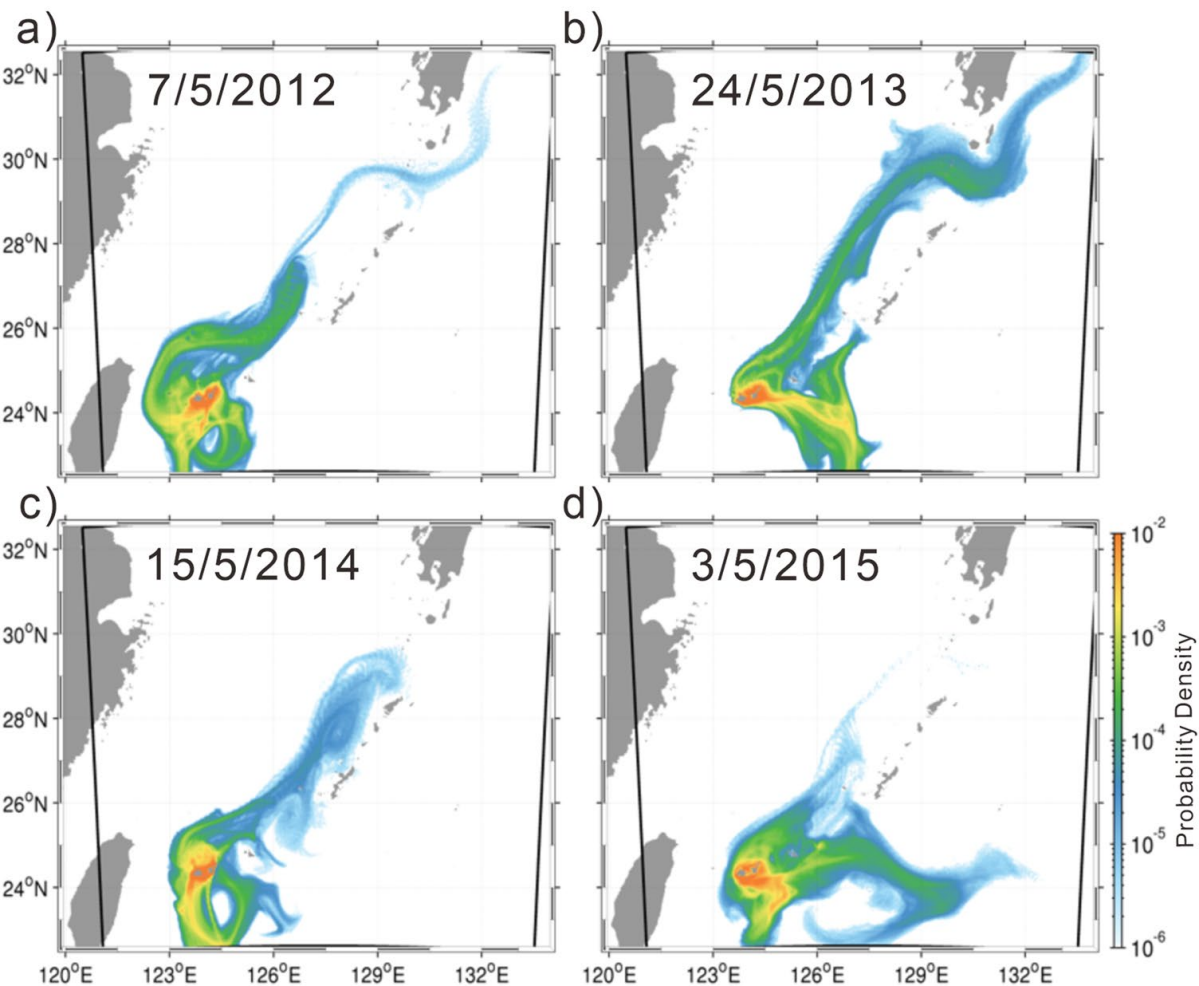
Probability density functions of reachability for particles discharged at 20:00 for 14 days beginning on the night after the full moon during a spring tide in May during the years 2012–2015. Warm colors indicate high probability densities. Advection time was 21 days in all cases, and discharge periods began on (a) 7 May 2012, (b) 24 May 2013, (c) 15 May 2014, and (d) 3 May 2015.

**Figure 9:**
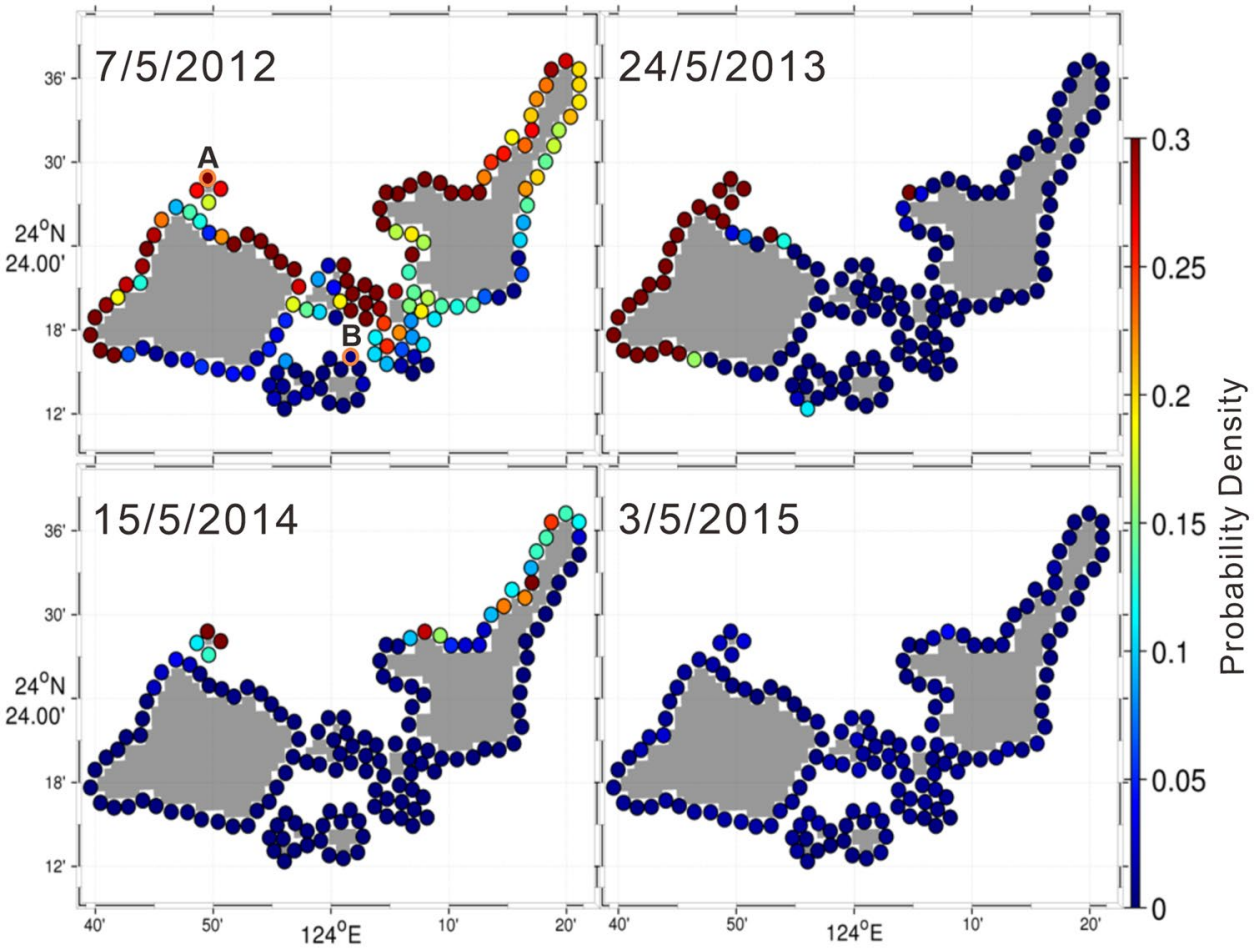
Probability density functions of reachability for long-distance particles during the years 2012–2015. The orange circles (marked (**A**,**B**)) show the northern coast of Hatoma Island and Urabishi reef, respectively.

**Figure 10:**
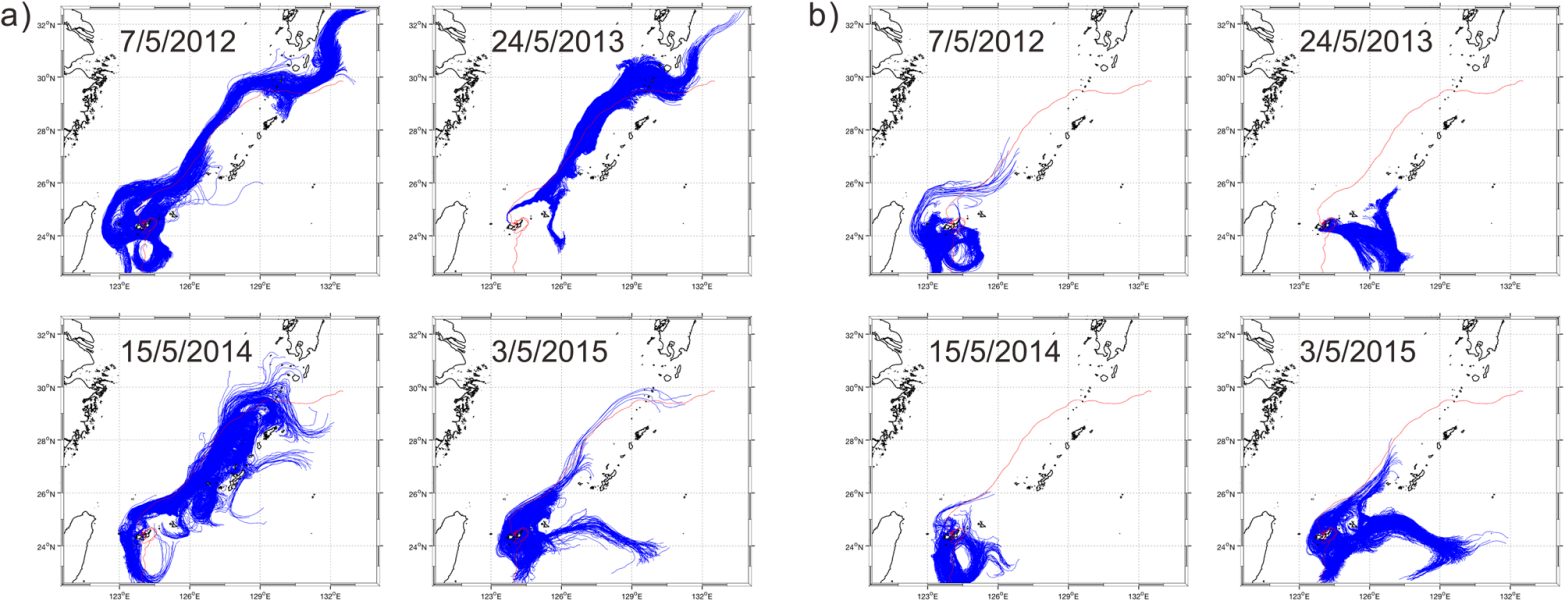
Trajectories of particles discharged from (**a**) the northern coast of Hatoma Island and (b) Urabishi reef during the years 2012–2015. Dates at the top of each map show the first particle discharge day in each year, and red lines indicate the trajectory of a drifter buoy observed during 2006 that followed the Kuroshio Current^4^.

We also sought to identify source areas around the Sekisei Lagoon for reefs in the Kuroshio downstream region and hence for aiding poleward coral habitat migration due to the influence of global climate change. To identify simulated particles that drifted from around the Sekisei Lagoon to the Kuroshio downstream region (including Okinawa Island) or otherwise drifted in a poleward direction, we focused on particles that were advected north of 26.5° N (hereafter referred to as long-distance particles [LDPs]). Many particles from the northern coasts of Iriomote and Ishigaki islands drifted to the Kuroshio downstream region (Fig. 8), as did particles from the northern coast of Hatoma Island (Fig. 9). This was not true for particles from southern coasts, such as the Urabishi reef (which suffered extensive damage from the 2016 bleaching event) (Figs. 9, 10).

## Discussion and concluding remarks

Our results reveal the location of source areas outside of the lagoon that could export coral spawn and larvae to the lagoon interior. This is an important process in the potential recovery of coral reefs in this area. Major source areas were located around Hatoma Island and the northern and northeastern coasts of Iriomote Island. Hatoma Island was also a major source area for simulated coral spawn and larvae arriving in the Kuroshio downstream region. In fact, it was the most important source area for both the interior Sekisei Lagoon and the Kuroshio downstream region. These results can be used to aid recovery of coral reefs in the entire coastal zone of the Sekisei Lagoon and the Kuroshio downstream region by informing the design of effective coral reef conservation areas in this region and directing efforts to remove *Acanthaster planci* (crown-of-thorns starfish) and transplant coral seedlings.

Previous research has highlighted the difficulty of tracking simulated coral spawn and larvae in complex generalized zonal current systems such as the eastern equatorial Pacific, where the North Equatorial Current, South Equatorial Current, and Northern Equatorial Countercurrent interact with prevailing easterlies under the influence of ENSO variability^12^. In contrast, our study region features a relatively simple and strong poleward western boundary current, the Kuroshio, which maintains a distinct surface current and never directs equatorward. Furthermore, the Kuroshio passes near the Coral Triangle in the further upstream region, which is known as the center of global coral reef biodiversity^13^. Accordingly, poleward coral mass migrations can occur relatively easily and can be tracked with relative simplicity in this region. The areas investigated in this study (the Sekisei Lagoon and Nansei Archipelago) play a key role as stepping stone relay stations in poleward migration mostly by the Kuroshio, thus the identification of coral spawn and larval sources in these areas is of crucial importance.

Given that global climate change is causing corals to migrate poleward, it is particularly important to conserve coral populations in upstream regions, especially in the areas that serve as sources of coral spawn and larvae in western boundary current systems. It is not a coincidence that upstream source areas in the western boundaries of subtropical gyres (i.e., where boundary currents direct poleward) around the world are located in warmer climates (as is true of the source areas identified in this study). Other examples of similar source regions include the Great Barrier Reef along the East Australian Current as well as reefs along the Gulf Stream, Agulhas Current, and Brazil Current. Identification and recovery of source areas in upstream regions of these western boundary currents should help coral populations in subtropical gyres adapt to global climate change by facilitating their poleward migration.
